# Reply to Xin Li and Yapeng Xing's Letter to the Editor re: Nielsen N.K, Hjort P.B, Hansen E.B, et al. Substaging and stratified treatment of T1 bladder cancer – a nationwide register-based study. Eur Urol Open Sci 2026;90:92-100

**DOI:** 10.1016/j.euros.2026.09.011

**Published:** 2026-10-20

**Authors:** Ninna K. Nielsen, Jørgen B. Jensen

**Affiliations:** a Department of Clinical Medicine, Aarhus University, Aarhus, Denmark; b Department of Urology, Aarhus University Hospital, Aarhus, Denmark; c Department of Urology, Gødstrup Regional Hospital, Gødstrup, Denmark; d Greenland Centre for Health Research, Institute for Health and Nature, Ilisimatusarfik/University of Greenland, Nuuk, Greenland

We thank the authors for their thoughtful comments on our nationwide study of pathological T1 substaging and substage-guided management of bladder cancer in Denmark and welcome the opportunity to further clarify the interpretation of our findings [1]. We agree with the important distinction between prognostic and treatment-predictive value. Our study demonstrated that pT1 substaging retained prognostic value after implementation into national clinical practice; it was not designed to establish pT1b as a predictive marker of benefit from immediate radical cystectomy.

We appreciate the authors for highlighting these methodological considerations, several of which also informed the interpretation and limitations of our study. As discussed in the article, treatment was guided by the substage under investigation, and the observed outcomes therefore reflect both tumor biology and management rather than the isolated effect of substaging. Accordingly, we stated that treatment selection limits causal interpretation. The recurrence and progression analyses were restricted to patients whose bladder was preserved, leaving a clinically selected pT1b population after 41% underwent early cystectomy; we acknowledged that this selection may exaggerate differences between pT1a and pT1b. Residual confounding from unavailable clinicopathological variables, including tumor size, multiplicity, concomitant carcinoma in situ, and lymphovascular invasion, was likewise described as a limitation.

Repeat resection is another important consideration. Repeat resection findings were unavailable in the registry, although a higher T1 substage recorded within 90 d resulted in reclassification, whereas ≥T2 disease identified within 90 d was considered initial understaging rather than progression. The 20% rate of muscle-invasive disease among patients with pT1b who underwent cystectomy therefore highlights a substantial risk of understaging. Detailed repeat-resection data would be important in future studies evaluating the independent value of pT1b for treatment selection.

The authors further raise the important question of whether pT1b modifies the benefit of immediate cystectomy compared with bladder-preserving treatment. We agree that prognostic differences alone cannot answer this question. Assessing treatment-effect heterogeneity, including a substage-by-treatment interaction among clinically comparable patients, would provide important additional evidence. Target trial emulation and evaluation of the incremental value of pT1b beyond established risk models are valuable approaches for future research but would require detailed information on treatment eligibility, repeat resection, tumor characteristics, and other determinants of treatment allocation that were unavailable in our registry.

The authors also highlight emerging computational pathology approaches. Because these were not evaluated in our study, their potential role in risk stratification or treatment selection is beyond the scope of the present analysis.

In conclusion, our findings support the prognostic relevance of pT1 substaging after nationwide implementation within a substage-guided management strategy but do not establish pT1b as a predictive marker of benefit from immediate cystectomy. We appreciate the authors’ emphasis on this distinction and the important directions they identify for future research.

  ***Conflicts of interest***: Jørgen Bjerggaard Jensen reports advisory board membership for Ferring, Roche, Cepheid, Urotech, Olympus, AMBU, Janssen, and Cystotech; speaker roles for Medac, Olympus, Photocure ASA, and Conmed; and research collaborations with Medac, Photocure ASA, Roche, Ferring, Olympus, Intuitive Surgery, Astellas, Cepheid, Nucleix, Urotech, Pfizer, AstraZeneca, MeqNordic, Laborie, OneMed, AMBU, and Cystotech. Ninna Kjær Nielsen has nothing to disclose.

  ***Funding support:*** This study was completed as part of a PhD funded by 10.13039/501100009708Novo Nordisk Fonden Project Grant in Surgical Research (reference NNF19OC0058534) and 10.13039/100032253Nordic Cancer Union Research Grant (reference A16003). The funding organizations had no role in the study design, conduct, data analysis, or interpretation.
